# Effects of a modular intervention on mobility and activities of daily living in geriatric patients in an acute hospital settings – results of the stepped-wedge cluster-randomized redurisk study

**DOI:** 10.1007/s40520-026-03349-9

**Published:** 2026-03-08

**Authors:** Rieka von der Warth, Boris A. Brühmann, Erik Farin-Glattacker, Felix Kentischer, Andy Maun, Christoph Maurer, Vitalii Minin, Alexander Ritzi, Claudia Salm, Sebastian Voigt-Radloff

**Affiliations:** 1 Section for Health Services Research and Rehabilitation Research, Institute for Medical Biometry and Statistics, Faculty of Medicine, Medical Center - University of Freiburg, University of Freiburg, Freiburg, Germany; 2 Center for Geriatrics and Gerontology, Medical Center - University of Freiburg, Freiburg, Germany; 3 Institute of General Practice/Family Medicine, Faculty of Medicine, Medical Center - University of Freiburg, University of Freiburg, Freiburg, Germany; 4 University of Bonn, University Hospital Bonn, Institute for Family Medicine, Bonn, Germany

**Keywords:** Frailty, Geriatric, Hospitalizations, Mobility, Risk-assessment

## Abstract

**Background:**

Hospitalization increases the risk of reduced mobility and diminished activities of daily living (ADL) in geriatric patients. Using a multimodal intervention including delirium prevention, mobility training, care planning, and polypharmacy management, the ReduRisk project aimed to mitigate these risks through a risk-adapted nursing approach.

**Aims:**

This paper seeks to describe the results of the multimodal ReduRisk study on the mobility and ADL in hospitalized geriatric patients. We hypothesized that patients in the intervention group will have better mobility and ADL than those in the control group.

**Methods:**

A monocentric, cluster-randomized trial enrolled a total of *N* = 589 participants. Clusters were based on different department at the University Hospital of Freiburg. Data collection occurred at three measurement points (hospital admission, discharge, and six months post-discharge) using the Barthel Index (ADL) and the Short Physical Performance Battery (SPPB, mobility). Multilevel analyses were conducted.

**Results:**

Of the *N* = 589 participants, 48% were female. Average age was 79.57 years (SD = 5.44 years). In the intervention group (IG), mobility significantly improved between t0 and t1 (β = 0.27; *p*<.01) and between t0 and t2 (β = 0.70; *p*<.001). Compared to the control group (CG), IG mobility improved significantly more from t0 to t2 (β=-0.30; *p*<.05). Regarding ADL, the IG also showed significant improvements between t0 and t1 (β = 3.69; *p*<.05) and t0 and t2 (β = 7.98; *p*<.001). Compared to the CG, IG participants experienced significantly more significant improvements (t0-t1: β=-6.93; *p*<.01; t0-t2: β=-8.40; *p*<.01).

**Discussion:**

The ReduRisk intervention led to moderate improvements in ADL and small gains in mobility. These findings support the importance of early and sustained mobility interventions in clinical care.

**Conclusions:**

We observed promising results for the risk-adjusted ReduRisk intervention in reducing health-related risks among geriatric patients.

**Trial registration:**

German Clinical Trials Register, DRKS00025594, date of registration 09/08/2021.

**Supplementary Information:**

The online version contains supplementary material available at 10.1007/s40520-026-03349-9.

## Background

Geriatric patients face an elevated risk of functional decline and deterioration in health status during hospital stays [1, 2]. This decline often manifests in reduced mobility, diminished activity of daily living, the onset of delirium, and increased multimorbidity. Consequently, the probability of discharge to a home environment decreases, while the risk of institutionalization in nursing homes or rehospitalizations increases [3–5]. Such deterioration is not always entirely attributable to the severity of the initial illness or pre-existing comorbidities; instead, it may also be associated with low levels of mobilization during hospital stays, as geriatric patients predominantly remain in bed [6, 7].

The ReduRisk study, funded by the Innovation Fund of the German Federal Joint Committee (G-BA), aimed to reduce these risks by implementing a risk-adapted intervention at the University Medical Center Freiburg [8]. An individualized intervention plan was developed based on a risk screening conducted upon hospital admission. The intervention included delirium prevention, mobility training, care planning, polypharmacy management, and health information provision [8]—all of which have been previously shown to be effective in reducing risks of immobility and decrease in ADL in similar contexts [9–13]. To our knowledge, ReduRisk is the first study aiming to reduce these risks simultaneously through a modular intervention. This modular intervention, led by nursing professionals, physiotherapists, health scientists, and physicians, was designed and implemented by an interprofessional team [8, 14]. The delirium prevention module was inspired by the AKTIVER program from the PAWEL study [13, 14]. During their hospital stay, patients were instructed in the individual modules, which were tailored to their specific risk profiles. The aim was to enable patients to perform the respective exercises independently after discharge.

The study’s primary goal was to improve mobility and activity of daily living (ADL) during hospitalization and sustain these effects six months post-discharge. We tested the following hypotheses:

1. Patients in the intervention group (IG) will have better mobility and ADL at discharge than those in the control group (CG).
2. Patients in the IG will have better mobility and ADL six months post-discharge compared to CG patients.

## Methods

The ReduRisk study received ethical approval from the Ethics Committee of the Albert Ludwig University of Freiburg (Approval No. 21-1240) and was prospectively registered in the German Clinical Trials Register (DRKS00025594). All participants provided written informed consent following a detailed explanation of the study. For participants under legal guardianship, the authorized representative provided consent. Reporting adhered to the CONSORT statement extension for stepped-wedge cluster-randomized trials [15].

### Study design

Considering feasibility, we conducted a stepped-wedge cluster-randomized design across six hospital departments (clusters) at the University Medical Center Freiburg. This approach allowed for phased implementation in each department, addressing potential implementation challenges. The intervention period lasted from October 2021 to March 2023, with departments included quarterly. Participant follow-up concluded in September 2023.

Participants aged 70 years or older were included if they showed increased risks for delirium, mobility restrictions, functional decline, or re-hospitalization during initial hospital screening and were patients in the participating departments. Exclusion criteria included acute delirium or end-stage palliative care. Screening for eligible patients was conducted daily by the intervention team, based on new admissions to the department. Information on recruitment, participating departments, and sample size calculation can be found in Online Supplement 1.

### Intervention

The intervention comprised five modules tailored to the individual risk profiles of the participants in the IG. Regardless of their risk profile, all participants in the IG were offered individualized health information. Additional modules were offered depending on their identified needs, including delirium prevention, mobility training, care planning, and polypharmacy management. Participants were allowed to decline single modules based on their preferences. The study staff administered the intervention for up to 10 days during the hospital stay. The study staff consisted of a doctor, nurses and physiotherapists. Participants were trained to continue applying the intervention measures independently for six months following discharge. To facilitate independence in the intervention, we allowed participants to use a tablet-based system. Those who chose this option received a tablet crash course as a sixth module. The control group (CG) received standard care based on their underlying diagnosis and did not receive any of the intervention modules. Standard care comprised guideline-conform responses to the occurrence of specific healthcare problems, such as physiotherapy sessions or clinic-specific delirium management protocols including adjustments to medication and closer monitoring by nursing staff up to one-to-one observation. Additional details regarding the intervention content and implementation process are available in Online Supplement 2 and the publication by Göhner et al. [8].

### Assessments

Blinded assessors recorded the primary outcomes at three measurement points: at hospital admission (t0), at discharge (t1, max. two weeks after admission), and six months post-discharge (t2). Assessments were conducted as structured self-report interviews with the participants. Assessments could be paused if interruptions occurred, for example due to diagnostic procedures or therapeutic appointments during hospitalization, or if the participant showed signs of fatigue. In cases of legal guardianship, assessments were conducted as proxy interviews. Data was collected using the REDCap platform [16, 17]. Patients who remained hospitalized for longer than two weeks still completed their T1 assessment after two weeks and were subsequently trained to conduct the intervention independently. Post-discharge assessments were conducted by the assessor at the participants’ homes whenever possible, or by telephone when an in-person visit was not feasible. While the assessors were blinded, neither the participants nor the intervention providers were blinded due to the group assignments.

We assessed mobility using the Short Physical Performance Battery (SPPB) [18–20]. This tool includes five exercises that measure balance, gait speed, and leg strength. The results are summed up to a Likert scale ranging from 0 to 12, where 12 represents excellent mobility. For intervention planning purposes, we categorized the scores into four levels according to Guralnik et al. [21]: participants scoring between 0 and 3 were considered bed-mobile, those scoring between 4 and 6 were classified as room-mobile, those scoring between 7 and 9 as ward-mobile, and those achieving scores between 10 and 12 were deemed not in need of mobility training.

ADL was measured using the Barthel Index [22]. This index captures the ability to perform ten basic tasks, with scores ranging from 0 to 100. Higher scores indicate greater independence in ADL. The Barthel Index was treated as an interval-scaled variable for the analyses, rather than being categorized as originally proposed by the authors.

Additional baseline information was collected at t0 via personal interview to examine potential influences on the primary outcomes. Information included the participant’s age, gender, department affiliation, and the number of falls experienced within the three months before hospital admission. The use of mobility aids and whether the participant underwent surgery during the current hospital stay were also recorded. Subjective pain levels were documented on an interval scale ranging from 1 to 10, and multimorbidity was evaluated based on the framework by Tooth et al. [23].

### Analyses

Initially, descriptive statistics were calculated for the primary outcomes, along with the effect size d, according to Morris [24]. This metric compares the effects between the intervention group (IG) and the control group (CG) within a pre-post design while accounting for any baseline differences between the groups at t0. We interpreted the effect sizes based on Cohen’s thresholds (Cohen, 1988): values between 0.1 and 0.3 were considered minor, 0.3 and 0.5 moderate, and values above 0.5 were classified as significant effects.

The analyses of the primary outcomes were conducted using multilevel modelling. For mobility, a negative binomial regression model was applied, while the analysis of ADL was conducted using a quantile multilevel regression. The three measurement time points on the first level were defined within individuals, while the second level represented the group assignment (IG vs. CG). Interactions between the two levels were evaluated using likelihood-ratio tests. To ensure robust results, the models included several covariates collected at baseline, including age, gender, department affiliation, number of falls before hospital admission, use of mobility aids, surgery during the current hospital stay, number of risks identified during risk screening, subjective pain levels, and multimorbidity. Unstandardized beta coefficients (β) were used to depict predicted changes in the outcome scores. We performed all analyses using R statistical software [25].

## Results

### Study participants

**Fig. 1:**
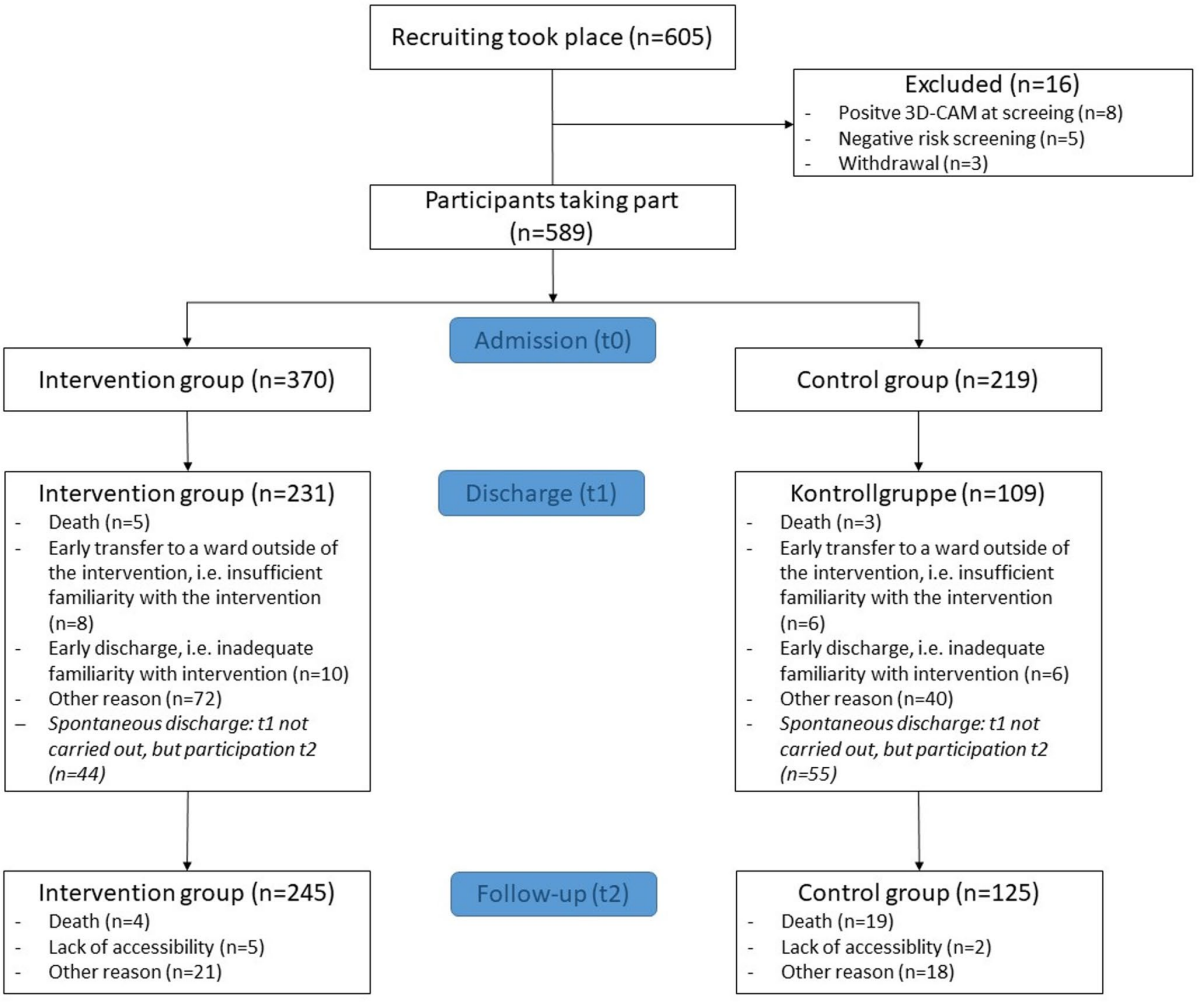
Flow Chart of the ReduRisk study

Overall, we were able to recruit *n* = 605 patients from the different wards based on inclusion criteria for the ReduRisk study. After screening, *n* = 589 participants were included in the ReduRisk study, with 370 assigned to the intervention group (IG) at baseline (t0). At discharge (t1), data were available for 340 participants. Six months post-discharge (t2), assessments were conducted with 370 participants. The increase in assessments at t2 can be explained by unplanned or early discharge, which prevented data collection at t1. Nevertheless, these patients were again assessed as t2 where possible. Figure 1 overviews the number of participants assessed at each time point.

Of the 589 participants, 306 (52%) were male. The gender distribution was skewed between the groups; in the control group (CG), 59.8% of participants were male, while in the IG, 52.7% were female. The average age of the participants was 79.57 years (± 5.44 years). Participants in the IG were statistically significant younger, with an average age of 79.05 years (± 5.29 years), compared to the CG (mean age 80.46 ± 5.56 years; *p* = .003). On average, we identified 3.52 (SD = 0.97) risks during risk screening in both groups. Participants in the intervention group received between two to five intervention modules. A total of *N* = 337 participants received individualized health information; *n* = 331 received delirium prevention, *n* = 306 mobility training, *n* = 166 care planning, and *n* = 141 polypharmacy management. Further sociodemographic and clinical characteristics of the study population are presented in Table 1.

**Table 1:** Descriptive statistics

		All participants *N* = 589	IG *N* = 370	CG *N* = 219
		*N* (%)	*N* (%)	*N* (%)
Gender				
	Female	283 (48.00)	195 (52.70)	88 (40.20)
	Male	306 (52.00)	175 (47.30)	131 (59.80)
Department				
	Orthopedics and Trauma Surgery	183 (31.10)	183 (49.50)	0 (0.00)
	General and Visceral Surgery	34 (5.80)	24 (6.50)	10 (4.60)
	Neurosurgery	131 (22.20)	91 (24.60)	40 (18.30)
	Internal Medicine II (Gastroenterology, Hepatology, Endocrinology, and Infectious Diseases)	70 (11.90)	20 (5.40)	50 (22.80)
	Neurology and Neurophysiology	122 (20.70)	46 (12.40)	76 (34.70)
	Nephrology and General Medicine	49 (8.30)	6 (1.60)	43 (19.60)
Mobility Aid Use				
	Yes	239 (40.60)	159 (48.30)	80 (42.10)
	No	280 (47.50)	170 (51.70)	110 (57.90)
Surgery during the Current Stay				
	Yes	327 (56.00)	273 (74.40)	54 (24.90)
	No	257 (44.00)	94 (25.60)	163 (75.10)
		**Mean (SD)**	**Mean (SD)**	**Mean (SD)**
Age (years)		79.57 (5.44)	79.05 (5.29)	80.46 (5.56)
Number of risks identified during risk screening		3.52 (0.97)	3.53 (0.94)	3.49 (1.02)
Falls in the 3 Months Before Hospitalization		2.14 (7.81)	1.75 (6.99)	2.73 (8.94)
Pain (0–10 scale)		2.67 (2.73)	2.98 (2.70)	2.15 (2.72)
Multimorbidity		4.43 (3.20)	4.05 (3.09)	5.05 (3.28)

IG = Intervention Group; CG = Control Group; SD = Standard Deviation

### Effects on mobility and ADL

In terms of the primary outcome of mobility, both the IG and the CG showed improvements from baseline (t0) through discharge (t1) and six months post-discharge (t2). In the IG, mobility increased from an average Short Physical Performance Battery (SPPB) score of 4.02 (± 4.00) at t0 to 7.33 (± 3.89) at t2. The CG’s mobility improved from 4.69 (± 4.05) at t0 to 6.88 (± 3.70) at t2.

Regarding ADL, as measured by the Barthel Index, the IG demonstrated improvements across all three measurement points. Scores increased from 77.63 (± 25.86) at t0 to 80.32 (± 22.87) at t1 and 89.59 (± 18.51) at t2. In the CG, however, there was a decline in ADL scores between t0 (84.97 ± 21.03) and t1 (81.88 ± 21.29), followed by an improvement to 88.12 (± 20.93) at t2. In the between-group comparison, effect sizes favoured the intervention group across all time intervals. For the SPPB, small effects were observed from T0 to T1 (*d* = 0.18) and from T1 to T2 (*d* = 0.10), while a cumulative effect emerged from T0 to T2 (*d* = 0.28). For the Barthel Index, a small effect was found from T0 to T1 (*d* = 0.24), a small-to-moderate effect from T0 to T2 (*d* = 0.36), and a small additional effect from T1 to T2 (d = 0.14). Detailed descriptive statistics for both the SPPB and the Barthel Index are presented in Table 2.

**Table 2:** Descriptive data for SPPB and Barthel index

SPPB										Effect size d sense Morris [24]		
	*T0*			*T1*			*T2*			*T0 vs. T1*	*T0 vs. T2*	*T1 vs. T2*
	*Mean*	*SD*	*Min-Max*	*Mean*	*SD*	*Min-Max*	*Mean*	*SD*	*Min-Max*			
IG	4.02	4.00	0.00–12.00	4.99	4.12	0.00–12.00	7.33	3.89	0.00–12.00	0.18	0.28	0.10
CG	4.69*	4.05	0.00–12.00	4.94	4.07	0.00–12.00	6.88	3.70	0.00–12.00			

Barthel-Index										Effect size d sense Morris [24]		
	*T0*			*T1*			*T2*			*T0 vs. T1*	*T0 vs. T2*	*T1 vs. T2*
	*Mean*	*SD*	*Min-Max*	*Mean*	*SD*	*Min-Max*	*Mean*	*SD*	*Min-Max*			
IG	77.63	25.86	0.00-100.00	80.32	22.87	0.00-100.00	89.59	18.51	20.00-100.00	0.24	0.36	0.14
CG	84.97	21.03	10.00-100.00	81.88	21.29	25.00-100.00	88.12*	20.93	10.00-100.00			

IG = Intervention Group; CG = Control Group; * <.05; *p* 0.05, significant between-group difference Categorization of SPPB: bed-mobile = 0 - 3; room-mobile = 4 - 6; ward-mobile = 7 - 9; no need of mobility training = 10 - 12

After controlling for baseline covariates, the IG showed significant improvements in mobility and ADL compared to the CG. For mobility, the IG improved significantly from t0 to t1 (β = 0.27;*p*<.01) and from t0 to t2 (β = 0.70;*p*<.001). Compared to the CG, the IG experienced a significantly greater improvement in mobility between t0 and t2 (β=−0.30;*p*<.05). Factors such as a low number of risk identified during risk screening positively influenced mobility (β=−0.26;*p*<.001), while the use of a mobility aid (β=−0.64;*p*<.001), greater pain levels (β=−0.02;*p*<.05), and higher multimorbidity (β=−0.04;*p*<.001) negatively impacted mobility. We found no significant effects of age, gender, department affiliation, falls, or surgery on mobility.

Regarding ADL, there were no significant differences between the IG and CG at baseline. Within the IG, ADL scores improved significantly from t0 to t1 (β = 3.69;*p*<.05) and from t0 to t2 (β = 7.98;*p*<.001). Compared to the CG, the IG experienced stronger ADL improvements from t0 to t1 (β=−6.93;*p*<.01) and from t0 to t2 (β=−8.40;*p*<.01). As with mobility, less risks identified during risk screening was associated with better ADL outcomes (β=−2.95;*p*<.05). Participants in the departments of General and Visceral Surgery (β = 7.78;*p*<.01) and Neurology and Neurophysiology (β = 7.07;*p*<.05) had significantly better ADL outcomes than those in Orthopedics and Trauma Surgery. Negative impacts on ADL were observed for mobility aid use (β=−10.65;*p*<.001), higher pain levels (β=−0.63;*p*<.05), and greater multimorbidity (β=−1.03;*p*<.001). As with mobility, no significant effects of age, gender, falls, or surgery on ADL were detected. Table 3 shows the results.

**Table 3:** Multilevel analyses of mobility and ADL

	Mobility	ADL
	β	β
*Outcome-related Analysis Variables*		
Change in IG between t0 and t1	0.27**	3.69*
Change in IG between t0 and t2	0.70***	7.98***
Difference IG vs. CG at t0	0.23*	3.65
Change in IG between t0 and t1 compared to CG	-0.18	-6.93**
Change in IG between t0 and t2 compared to CG	-0.30*	-8.40**
*Intervention-related Covariates*		
Number of risks identified during risk screening	-0.26***	-2.95*
Department *(Intercept = Orthopaedics and Trauma Surgery)*		
General and Visceral Surgery	-0.08	7.78**
Neurosurgery	-0.02	2.50
Internal Medicine II (Gastroenterology, Hepatology, Endocrinology, and Infectious Diseases)	-0.21	4.76
Neurology and Neurophysiology	-0.04	7.07*
Nephrology and General Medicine	-0.17	3.99
*Sociodemographic Covariates*		
Age	-0.01	-0.11
Gender (*Intercept=female*)	0.08	1.52
*Health-related Covariates*		
Falls at t0	< 0.01	0.10
Mobility aid at t0 *(Intercept = no)*	-0.64***	-10.65***
Surgery during hospital stay *(Intercept = yes)*	-0.02	-2.03
Pain	-0.02	-0.63*
Multimorbidity	-0.04***	-1.03***

IG = Intervention Group; CG = Control Group, β = unstandardized Beta

*p<.05; **p<.01; ***p<.001

## Discussion

The ReduRisk study investigated a nurse-led, risk-adjusted modular intervention for geriatric patients during and after hospitalization. The ReduRisk intervention built on established interventions, particularly in delirium prevention [13] and mobility training [9]. However, its innovative contribution lies in its multimodular structure, allowing multiple risks to be addressed simultaneously in a risk-adapted manner. Overall, we found minor effects on mobility and moderate effects on ADL, which must be discussed on a clinical level, even though we could confirm our hypotheses.

Regarding mobility, the IG showed a more significant improvement over the six-month follow-up period than the CG. According to Guralnik et al. [21], participants in the CG remained in the “room-mobile” category, while those in the IG improved from “bed-mobile” to “ward-mobile.” These findings suggest that the multimodal ReduRisk intervention had more beneficial effects than previously reported in the literature, where mobility training alone has shown a limited impact on the mobility and functionality of patients [11]. However, it is important to note that both groups had SPPB scores below 10 even six months post-discharge. This score is still associated with higher mortality rates [26]. This emphasizes the need for ongoing efforts to improve patient mobility. Thus, we support the call for mobility training during hospital stays and after discharge. The initial steps toward developing joint recommendations for such training should be further explored [27, 28].

The intervention’s impact on ADL was more pronounced when comparing the IG to the CG. While the CG experienced a reduction in ADL during hospitalization, the IG demonstrated improvements at discharge and six months post-discharge. All changes between the IG and CG were statistically with moderate effect sizes indicating clinical relevance. Results were influenced by mobility aid use, pain levels and greater multimorbidity. This result suggests that the intervention successfully prevented the typical decline in ADL in hospitalized patients [2]. Previous studies have confirmed that geriatric interventions can at least avoid a reduction in ADL [10]. The results of the ReduRisk study align with international findings, indicating that the intervention positively impacted ADL.

### Strength and limitations

One of the strengths of the ReduRisk study is its focus on addressing multiple risks simultaneously. Additionally, the study had a relatively long follow-up period of six months, whereas similar studies typically conduct follow-up assessments only within a few weeks [10].

Another strength is the individualized approach of the intervention, which was tailored based on a risk screening conducted at the time of hospital admission. This individualized risk-adapted intervention has been recognized in the international literature as one of the most effective strategies for reducing the health risks of geriatric patients [1]. According to our analyses, the study also successfully integrated the intervention into routine care as part of a process evaluation [29], which may be directly linked to the positive outcomes observed. Nevertheless, data on patients’ adherence after discharge are limited. As we were unable to assess or account for its potential influence on study outcomes, this may have introduced an unmeasured bias in the results at t2. Therefore, the findings should be interpreted with caution. Future studies should focus on strategies to enhance and systematically assess intervention adherence after discharge.

Despite these strengths, the study does have some limitations. For example, although cluster randomization was used, there were baseline differences between the IG and CG regarding the primary outcomes. The IG participants showed higher mobility and ADL burdens at the start of the study. Although we controlled for these differences in the analyses, it remains unclear how the IG would have performed if they had been more comparable to the CG at baseline. Furthermore, we have an imbalance in group size between the clusters. Variation in cluster sizes can be attributed to differences in patients’ disease burden and length of stay across clusters, which substantially complicated recruitment for the ReduRisk study. Therefore, assumptions for cluster-randomized studies are violated, limiting the interpretability of the results. Lastly, it is impossible to determine which specific module or combination of modules contributed most to the improvements observed. Furthermore, a substantial number of participants were discharged before the T1 assessment, which may have introduced bias. Whenever possible, these participants were included in the T2 assessment.

## Conclusion

Overall, the ReduRisk study demonstrated promising results for reducing decrease in mobility and ADL in geriatric patients. Although analyses of routine data regarding healthcare utilization, rehospitalization, and institutionalization are still pending, the intervention may offer valuable benefits for geriatric patients’ health.

## Supplementary Information

Below is the link to the electronic supplementary material.

Supplementary Material 1

Supplementary Material 2

## Data Availability

No datasets were generated or analysed during the current study.
