# Supplementary material for: Effects of a modular intervention on mobility and activities of daily living in geriatric patients in an acute hospital settings – results of the stepped-wedge cluster-randomized redurisk study
Source: Aging Clin Exp Res. 2026 Mar 8;38(1):100. doi: 10.1007/s40520-026-03349-9 (PMC13005855; doi:10.1007/s40520-026-03349-9)
Supplement: Supplementary file 1 — Supplementary Material 1 [file 40520_2026_3349_MOESM1_ESM.pdf]

Title - Effects of a Modular Intervention on Mobility and Activities of Daily Living in Geriatric Patients in an Acute Hospital Settings; Journal - Aging Clinical and Experimental Research; Author - Rieka von der Warth; Affiliation - Section for Health Services Research and Rehabilitation Research, Institute for Medical Biometry and Statistics, Medical Center - University of Freiburg, Faculty of Medicine, University of Freiburg, Freiburg, Germany; Corresponding author - boris.bruehmann@uniklinik-freiburg.de

Online Supplement 1 – Recruitment Procedure and Sample Size Calculation

Table 1 provides an overview of the planned recruitment numbers in the participating departments of the University Medical Center Freiburg. The six departments were included in the intervention every quarter. Before that, recruitment for the control group had already taken place in the respective departments. The order of inclusion in the intervention was determined by the institute conducting the independent evaluation using computer-generated randomization.

Table 1: Planned sample size and recruitment departments

Year	2021	2022				2023
Month	10-12	1-3	4-6	7-9	10-12	1-3
Intervention group, N=357	17	34	51	68	85	102
Orthopedics and Trauma Surgery	17	17	17	17	17	17
General and Visceral Surgery	17	17	17	17	17	17
Neurosurgery	17	17	17	17	17	17
Internal Medicine II (Gastroenterology, Hepatology, Endocrinology, and Infectious Diseases)	17	17	17	17	17	17
Neurology and Neurophysiology	17	17	17	17	17	17
Nephrology and General Medicine	17	17	17	17	17	17
Control group, N=255	85	68	51	34	17	

Regarding the sample size, the following considerations were made: The hypothesis of the superiority of the intervention group over the control group concerning the two primary endpoints, mobility and ADL functions (significance of the mean difference), was to be tested confirmatory. Based on Martínez-Velilla et al. [1], medium effect sizes were expected (assumption here: Cohen's d = 0.50). A Bonferroni adjustment was applied for sample size calculation for the alpha level due to the multiple testing of two primary endpoints (5% / 2 = 2.5%). An achievable sample size of N=17 per cluster and recruitment quarter was assumed, with a 10% dropout at t1 and another 10% at t2. This meant that N=13 cases per cluster and quarter with complete data would remain.

As part of the recruitment process, the interventionists identified potential participants daily based on new hospital admissions and the predefined inclusion criteria. These individuals were informed about the ReduRisk study, supported by study materials, and asked for their general willingness to participate. After obtaining written consent to participate in the study, a structured risk screening was conducted. The following cut-off values applied for inclusion in the study:

- Risk of functional decline and rehospitalization: ISAR (0 = good to 6 = poor) [2]. Cut-off value: ISAR ≥ 3 or 'yes' for ≥ 6 potentially inappropriate medications.
- Risk of immobility, falls, frailty, rehospitalization, and mortality: SPPB (12 = good to 0 = poor) [3–5]; Cut-off value: SPPB ≤ 9.
- Risk of delirium: as defined in the University Medical Center Freiburg guidelines for the prevention and treatment of delirium (UKF-Delirium-Standard) and the "3D-CAM." Patients with ≥ 1 risk factor are included in the study; however, patients with acute delirium are excluded, as they cannot provide informed consent [6].

If at least one cut-off value was reached, the person was included in the study.

Title - Effects of a Modular Intervention on Mobility and Activities of Daily Living in Geriatric Patients in an Acute Hospital Settings; Journal - Aging Clinical and Experimental Research; Author - Rieka von der Warth; Affiliation - Section for Health Services Research and Rehabilitation Research, Institute for Medical Biometry and Statistics, Medical Center - University of Freiburg, Faculty of Medicine, University of Freiburg, Freiburg, Germany; Corresponding author - boris.bruehmann@uniklinik-freiburg.de

References

1. Martínez-Velilla N, Casas-Herrero A, Zambom-Ferraresi F et al. Effect of Exercise Intervention on Functional Decline in Very Elderly Patients During Acute Hospitalization: A Randomized Clinical Trial. JAMA Intern Med. 2019; 179: 28–36. DOI: 10.1001/jamainternmed.2018.4869.
2. Singler K, Heppner HJ, Skutetzky A, Sieber C, Christ M, Thiem U. Predictive validity of the identification of seniors at risk screening tool in a German emergency department setting. Gerontology. 2014; 60: 413–9. DOI: 10.1159/000358825.
3. Volpato S, Cavalieri M, Sioulis F et al. Predictive value of the Short Physical Performance Battery following hospitalization in older patients. J Gerontol A Biol Sci Med Sci. 2011; 66: 89–96. DOI: 10.1093/gerona/glq167.
4. Veronese N, Bolzetta F, Toffanello ED et al. Association between Short Physical Performance Battery and falls in older people: the Progetto Veneto Anziani Study. Rejuvenation Res. 2014; 17: 276–84. DOI: 10.1089/rej.2013.1491.
5. Pavasini R, Guralnik J, Brown JC et al. Short Physical Performance Battery and all-cause mortality: systematic review and meta-analysis. BMC Med. 2016; 14: 215. DOI: 10.1186/s12916-016-0763-7.
6. Olbert M, Eckert S, Mörgeli R, Marcantonio E, Spies C. 3D-CAM Guideline-Conform Translation for German-Speaking Countries [3D-CAM: Delir-Testinstrument für deutschsprachigen Raum übersetzt]. Anasthesiol Intensivmed Notfallmed Schmerzther 2018; 53: 793–6. ger. DOI: 10.1055/a-0627-4601.
