# Supplementary material for: Effects of a modular intervention on mobility and activities of daily living in geriatric patients in an acute hospital settings – results of the stepped-wedge cluster-randomized redurisk study
Source: Aging Clin Exp Res. 2026 Mar 8;38(1):100. doi: 10.1007/s40520-026-03349-9 (PMC13005855; doi:10.1007/s40520-026-03349-9)
Supplement: Supplementary file 2 — Supplementary Material 2 [file 40520_2026_3349_MOESM2_ESM.pdf]

Title - Effects of a Modular Intervention on Mobility and Activities of Daily Living in Geriatric Patients in an Acute Hospital Settings; Journal - Aging Clinical and Experimental Research; Author - Rieka von der Warth; Affiliation - Section for Health Services Research and Rehabilitation Research, Institute for Medical Biometry and Statistics, Medical Center - University of Freiburg, Faculty of Medicine, University of Freiburg, Freiburg, Germany; Corresponding author - boris.bruehmann@uniklinik-freiburg.de

Online Supplement 2 – Information on the Intervention Contents

Intervention

The intervention consisted of six modules offered to study participants in the intervention group depending on their risk screening. Participants could choose between a tablet-based or paper-based implementation of the modules. If a participant preferred tablet-based implementation for at least one of the digitally feasible modules, an age-appropriate tablet with the ReduRisk app was provided free of charge for the intervention period. These devices featured a simplified user interface and easily readable text.

During the inpatient intervention phase, all study participants received the ReduRisk program for up to three 30-minute sessions daily. The intervention could be carried out for up to 10 days during the inpatient stay. Figure 1 provides an overview of the modules and their timeline.

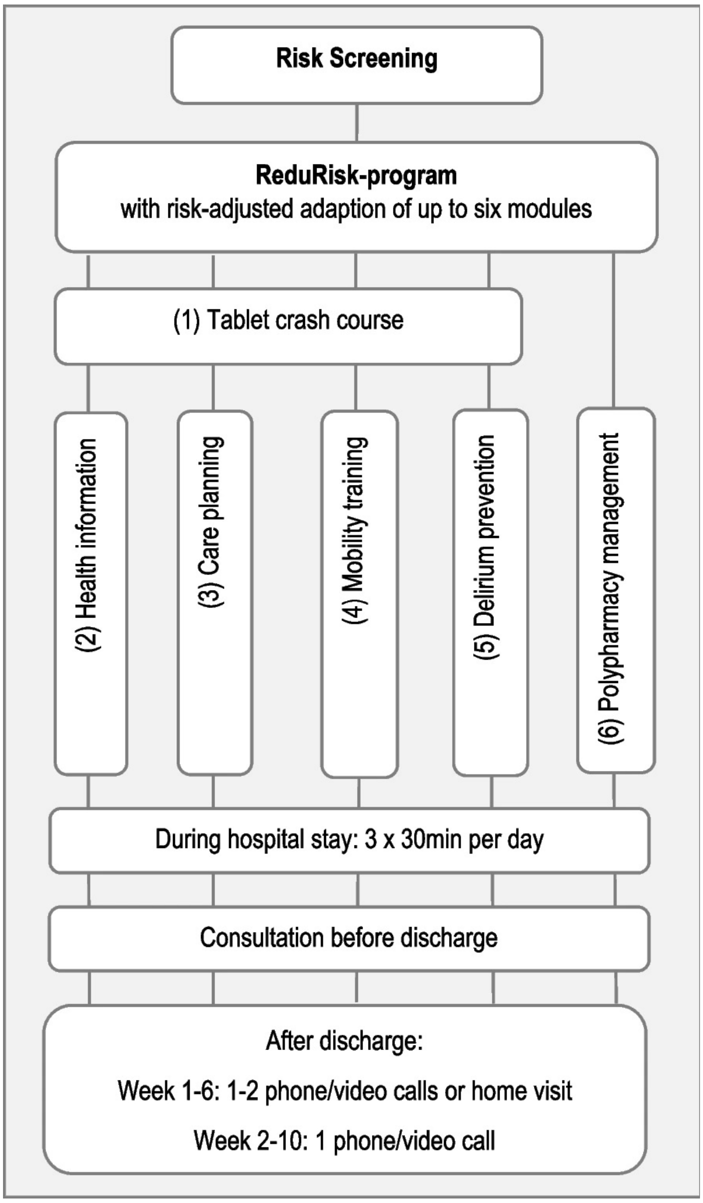

Figure 1: Intervention process, previously published in Göhner et al. [1]

Title - Effects of a Modular Intervention on Mobility and Activities of Daily Living in Geriatric Patients in an Acute Hospital Settings; Journal - Aging Clinical and Experimental Research; Author - Rieka von der Warth; Affiliation - Section for Health Services Research and Rehabilitation Research, Institute for Medical Biometry and Statistics, Medical Center - University of Freiburg, Faculty of Medicine, University of Freiburg, Freiburg, Germany; Corresponding author - boris.bruehmann@uniklinik-freiburg.de

Module

Tablet-Crash course

Triggered by a preference for using a tablet in at least one intervention module, this course aimed to build confidence in tablet usage. Interested participants received a 30–to 60-minute crash course explaining the tablet's essential functions and the features of the ReduRisk intervention. Participants also received a paper document illustrating essential tablet functions and controls using images, icons, and text. The content was customised to individual experience levels. Interventionists provided an overview of the intervention elements triggered by risk screening and determined which parts of the ReduRisk program would be implemented via tablet or paper. Participants requiring further assistance during their stay received ongoing support in tablet usage.

Personalised Health Information

This module provided all participants with information to enhance health and problem-solving competencies. Information was delivered via tablet or health binder upon request. It included health guidelines and patient guidelines from organizations such as AWMF, ÄZQ, and IQWIG, along with a communication guide for consultations with physicians. In the planning process, we drew on the projects LoChro [2], GAP [3], MedAlt [4], and PREPARE [5].

The thematic needs were identified based on the patient's medical record, t0-assessment results, and risk screening. Initial suggestions were discussed with participants and adjusted throughout the intervention. During the inpatient phase, topics included personal care, well-being, physical activity, emergency preparedness, and household management. Additional needs were assessed and addressed during post-discharge follow-up.

Delirium Prevention

The goal of this module was to prevent delirium during hospitalization. It was triggered by one or more predisposing risk factors identified through risk screening based on UKF standards.

Following the University Medical Center Freiburg standard, predisposing risk factors were recorded during the risk screening, and a personalized delirium risk profile was created during the first intervention contact. The intervention team implemented the resulting preventive measures daily throughout the hospital stay. The frequency and content of these measures were tailored to the individual risk profile and the participant's preferences.

Preventive measures were carried out in the areas of orientation, activation, mobilization, sleep promotion and relaxation, diagnostic assistance, and meal accompaniment. These measures were based on the multi-component delirium prevention program AKTIVER, which we implemented at the University Medical Center Freiburg in 2018 as part of the PAWEL consortium. The PAWEL study demonstrated a 33% reduction in delirium incidence in elective orthopaedic and abdominal surgeries [6].

After discharge, no further measures for delirium prevention were initiated by the interventionists. However, tablet participants could independently use the memory training application provided on the device and access additional resources, including a video and information on delirium.

Title - Effects of a Modular Intervention on Mobility and Activities of Daily Living in Geriatric Patients in an Acute Hospital Settings; Journal - Aging Clinical and Experimental Research; Author - Rieka von der Warth; Affiliation - Section for Health Services Research and Rehabilitation Research, Institute for Medical Biometry and Statistics, Medical Center - University of Freiburg, Faculty of Medicine, University of Freiburg, Freiburg, Germany; Corresponding author - boris.bruehmann@uniklinik-freiburg.de

Mobility training

Triggered by ISAR or SPPB assessment cut-off scores (≥3 for ISAR or ≤9 for SPPB), this module aimed to improve physical functionality.

Adapted from Martínez-Velilla's protocol [7], it included strength, balance, flexibility, cardiovascular exercises, and walking drills with increasing intensity levels [8–11].

Participants were grouped based on their mobility levels:

- Bed Mobility (SPPB 0-3): Strength, flexibility, and cardiovascular training.
- Room Mobility (SPPB 4-6): Strength, balance, flexibility, cardiovascular training, and assisted walking exercises.
- Unit Mobility (SPPB 7-12): Strength, balance, flexibility, cardiovascular training, and hallway walking exercises.

Each 30-minute mobility training session included 10 minutes for preparation and follow-up activities such as motivation, safety precautions, and education for participants and their caregivers, along with 20 minutes of exercises conducted either in the patient's room or a nearby designated area.

The interventionists tailored the exercises based on the participant's initial training needs and preferences, progressively increasing intensity throughout the intervention. Adjustments were made by modifying the exercise selection or increasing intensity, for example, by adding weights. All exercises were conducted under the supervision of trained interventionists.

When deemed safe, the training was gradually transitioned to self-guided exercises, with family members assisting as needed. For participants using tablets, exercise videos were provided, while those opting for paper-based intervention received printed instructions with visual aids.

Care Planning

If the ISAR score exceeded the cut-off value of ≥3 or the question "six or more medications" was answered with "yes," the Care Planning module was triggered. Analyzing informational needs and care-related issues served as the basis for creating the care plan. Additional informational needs were regularly assessed during care planning, and necessary information was provided.

In cases of polypharmacy, the care plan focused, among other things, on maintaining a complete and up-to-date medication list. During the hospital stay, solutions were developed collaboratively to prevent potential challenges and obstacles after discharge. The interventionists guided participants step by step in describing the care problem/goal and formulating corresponding solutions.

For participants opting out of tablet use, a structured form was provided, which could be stored in a health binder.

Polypharmacy Management

The Polypharmacy Management module was applied when participants took six or more medications and was conducted by a general medicine specialist (study physician).

Title - Effects of a Modular Intervention on Mobility and Activities of Daily Living in Geriatric Patients in an Acute Hospital Settings; Journal - Aging Clinical and Experimental Research; Author - Rieka von der Warth; Affiliation - Section for Health Services Research and Rehabilitation Research, Institute for Medical Biometry and Statistics, Medical Center - University of Freiburg, Faculty of Medicine, University of Freiburg, Freiburg, Germany; Corresponding author - boris.bruehmann@uniklinik-freiburg.de

During the inpatient intervention phase, the study physician prepared a medication recommendation and shared it with the responsible ward physicians. This recommendation was based on a prompt analysis of pre-existing and current medications shortly after hospital admission, considering the diagnoses recorded in the hospital's internal system, pre-existing conditions captured in the t0 assessment, and a personal consultation between the study physician and the participant.

The report included information about pre-existing conditions not yet documented in the hospital's system and recommendations for the current medication regimen based on the STOPP-START version 2 and Medication Appropriateness Index (MAI) criteria. The ward physicians could consult with the study physician during the hospital stay, and the recommendations could be adjusted anytime. The ward physicians were encouraged to include the ReduRisk medication recommendation text in the discharge letter to make it available to the outpatient care physicians.

Participants were also provided a letter addressed to their general practitioner (GP) and a confidentiality release form, offering a free service to prepare a polypharmacy recommendation for outpatient medication within 10 weeks after discharge. If the GP was interested, they could submit the participant's current medication and diagnosis lists to the study physician, who would analyze the diagnoses, medications, and supplementary data to produce another report for the GP. If inappropriate medication use was identified, the GP could also request a telephone consultation regarding polypharmacy.

Title - Effects of a Modular Intervention on Mobility and Activities of Daily Living in Geriatric Patients in an Acute Hospital Settings; Journal - Aging Clinical and Experimental Research; Author - Rieka von der Warth; Affiliation - Section for Health Services Research and Rehabilitation Research, Institute for Medical Biometry and Statistics, Medical Center - University of Freiburg, Faculty of Medicine, University of Freiburg, Freiburg, Germany; Corresponding author - boris.bruehmann@uniklinik-freiburg.de

References

1. Göhner A, Dreher E, Kentischer F et al. Reduction of care-relevant risks to older patients during and after acute hospital care (ReduRisk) - study protocol of a cluster randomized efficacy trial in a stepped wedge design. BMC Geriatr. 2022; 22: 754. DOI: 10.1186/s12877-022-03442-4.
2. Frank F, Bjerregaard F, Bengel J et al. Local, collaborative, stepped and personalised care management for older people with chronic diseases (LoChro): study protocol of a randomised comparative effectiveness trial. BMC Geriatr 2019; 19: 64. DOI: 10.1186/s12877-019-1088-0.
3. Voigt-Radloff S, Schöpf AC, Boeker M et al. Well informed physician-patient communication in consultations on back pain – study protocol of the cluster randomized GAP trial. BMC Family Practice 2019; 20: 33. DOI: 10.1186/s12875-019-0925-8.
4. Brefka S, Dallmeier D, Mühlbauer V et al. A Proposal for the Retrospective Identification and Categorization of Older People With Functional Impairments in Scientific Studies—Recommendations of the Medication and Quality of Life in Frail Older Persons (MedQoL) Research Group. Journal of the American Medical Directors Association 2019; 20: 138–46. DOI: 10.1016/j.jamda.2018.11.008.
5. Sudore RL, Boscardin J, Feuz MA, McMahan RD, Katen MT, Barnes DE. Effect of the PREPARE Website vs an Easy-to-Read Advance Directive on Advance Care Planning Documentation and Engagement Among Veterans: A Randomized Clinical Trial. JAMA Intern Med. 2017; 177: 1102–9. DOI: 10.1001/jamainternmed.2017.1607.
6. Deeken F, Sánchez A, Rapp MA et al. Outcomes of a Delirium Prevention Program in Older Persons After Elective Surgery: A Stepped-Wedge Cluster Randomized Clinical Trial. JAMA Surg. 2022; 157: e216370. DOI: 10.1001/jamasurg.2021.6370.
7. Martínez-Velilla N, Casas-Herrero A, Zambom-Ferraresi F et al. Effect of Exercise Intervention on Functional Decline in Very Elderly Patients During Acute Hospitalization: A Randomized Clinical Trial. JAMA Intern Med. 2019; 179: 28–36. DOI: 10.1001/jamainternmed.2018.4869.
8. Smart DA, Dermody G, Coronado ME, Wilson M. Mobility Programs for the Hospitalized Older Adult: A Scoping Review. Gerontol Geriatr Med. 2018; 4: 2333721418808146. DOI: 10.1177/2333721418808146.
9. Liu C-J, Chang W-P, Araujo de Carvalho I, Savage KEL, Radford LW, Amuthavalli Thiyagarajan J. Effects of physical exercise in older adults with reduced physical capacity: meta-analysis of resistance exercise and multimodal exercise. Int J Rehabil Res. 2017; 40: 303–14. DOI: 10.1097/MRR.0000000000000249.
10. Howe TE, Rochester L, Neil F, Skelton DA, Ballinger C. Exercise for improving balance in older people. Cochrane Database Syst Rev. 2011; 2011: CD004963. DOI: 10.1002/14651858.CD004963.pub3.
11. Heimbach B, Frings L, Dreikorn M et al. Effekte der wohnortnahen Ambulanten Geriatrischen Rehabilitation auf Mobilität und Selbstversorgungsfähigkeit. Frankfurt am Main; September 2015.
